# Epigenetic Drivers of Chemoresistance in Nucleobase and Nucleoside Analog Therapies

**DOI:** 10.3390/biology14070838

**Published:** 2025-07-09

**Authors:** John Kaszycki, Minji Kim

**Affiliations:** 1 Department of Biological Sciences, University of Connecticut, Storrs, CT 06269, USA; john.kaszycki@uconn.edu; 2 School of Pharmacy, University of Connecticut, Storrs, CT 06269, USA

**Keywords:** epigenetics, chemoresistance, DNA repair, histone modifications, translesion synthesis, base excision repair, autophagy

## Abstract

Nucleobase and nucleoside analogs are critical components of antimetabolite chemotherapy treatments used to disrupt DNA replication and induce apoptosis in rapidly proliferating cancer cells. However, the development of resistance to these agents remains a major clinical challenge. This review explores the epigenetic mechanisms that contribute to acquired chemoresistance, focusing on DNA methylation, histone modifications, and non-coding RNAs (ncRNAs). These epigenetic alterations regulate key processes such as DNA repair, drug metabolism, cell transport, and autophagy, enabling cancer cells to survive and resist therapeutic pressure. We highlight how dysregulation of DNA methyltransferases (DNMTs) and histone acetyltransferases (HATs) modulates expression of transporters (e.g., hENT1, *ABCB1*), DNA repair enzymes (e.g., Polβ, BRCA1/2), and autophagy-related genes (e.g., *CSNK2A1*, *BNIP3*). Furthermore, emerging roles for long non-coding RNAs (lncRNAs) and circular RNAs (circRNAs) in regulating nucleoside export and DNA damage response pathways underscore their relevance as therapeutic targets. The interplay of these epigenetic modifications drives resistance to agents such as gemcitabine and 5-fluorouracil across multiple tumor types. We also discuss recent progress in therapeutic interventions, including DNMT and HDAC inhibitors, RNA-based therapeutics, and CRISPR-based epigenome editing.

## Simple Summary

Cancer treatments that use drugs resembling DNA building blocks, known as nucleotide analogs, that insert themselves into DNA and prevent replication often stop working over time because cancer cells become resistant. This review explains how reversible epigenetic changes help cancer cells avoid being killed by these drugs. These changes do not alter the DNA sequence but can turn important genes on or off. We describe how cancer cells use these mechanisms to repair damaged DNA, pump drugs out of the cell, or break them down. We highlight the role of DNA packaging proteins (histones), small RNA molecules, and chemical tags on DNA that control how genes behave. Understanding these changes is important because it may allow clinicians to resensitize tumors and make them sensitive to treatment again and prevent relapse. We also explore new therapies, including drugs and gene editing tools, that target these epigenetic changes to improve the success of chemotherapy. These findings could lead to better outcomes for patients with difficult-to-treat cancers, especially those with pancreatic, colorectal, or breast cancer.

## 1. Introduction

**Figure 1:**
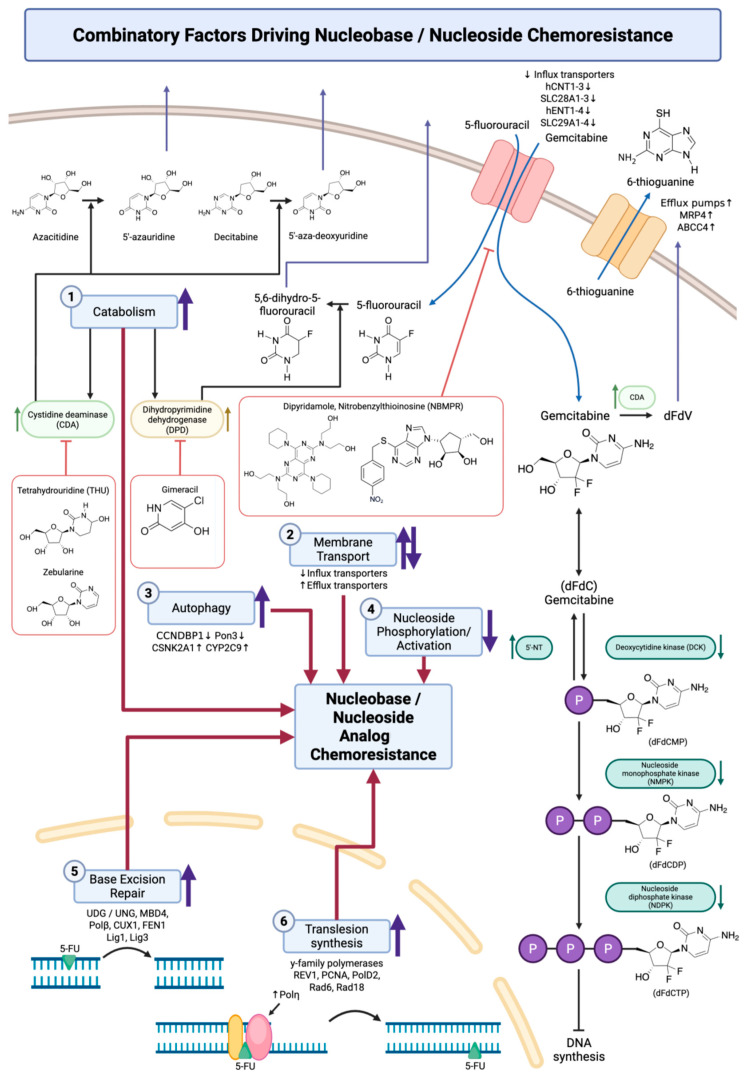
Combinatory mechanisms contributing to nucleobase/nucleoside analog chemoresistance. Multiple mechanisms collectively contribute to chemoresistance against nucleobase and nucleoside analogs, which are commonly used in cancer therapy. (1) Catabolism of agents such as 5-fluorouracil (5-FU), gemcitabine, and 6-thioguanine is mediated by enzymes including dihydropyrimidine dehydrogenase (DPD) and cytidine deaminase (CDA), limiting drug availability. Inhibitors like gimeracil and tetrahydrouridine (THU) can suppress these pathways. (2) Membrane transport influences drug influx and efflux, with downregulation of transporters (e.g., hCNT1-3, hENT1-4) and upregulation of efflux pumps (e.g., MRP4, ABCC4) reducing intracellular drug concentrations. (3) Autophagy-related genes (e.g., *CCNDBP1*, *Pon3*, *CSNK2A1*, *CYP2C9*) have been implicated in resistance by promoting drug degradation or survival under stress. (4) Nucleoside phosphorylation and activation involves stepwise phosphorylation by kinases (dCK, NMPK, NDPK), generating active triphosphate forms necessary for DNA incorporation. (5) Base excision repair (BER) enzymes (e.g., UDG, MBD4, Polβ, LIG1) remove nucleoside analogs misincorporated into DNA, reducing cytotoxic efficacy. (6) Translesion synthesis (TLS) allows bypass of drug-induced DNA lesions by specialized polymerases (e.g., REV1, Polη), preserving cell viability despite genotoxic stress. These interconnected resistance mechanisms hinder the therapeutic efficacy of nucleobase/nucleoside analogs. Created with BioRender.com (web application, accessed 6 May 2025).

The intricate mechanisms governing the development of chemoresistance to nucleobase and nucleoside analogs are fundamental to the development of cancer therapeutics. These drugs mimic endogenous purines or pyrimidines and interfere with DNA/RNA synthesis, leading to cell cycle arrest and apoptosis. Acquired chemoresistance to nucleoside analogs results in the relapse and progression of cancer. Studying the epigenetic factors that contribute to nucleobase/nucleoside analogs is particularly interesting because cytosine analogs are DNA methyltransferase inhibitors (DMNTis) and therefore alter the epigenetic landscape contributing to their own resistance. Among the pathways that contribute to chemoresistance of such analogs, translesion synthesis (TLS) and base excision repair (BER) are the most fundamental [1]. These DNA damage tolerance pathways directly counteract the effects of the insertion of nucleobase/nucleoside analog-derived nucleotides inserted into DNA. TLS is a pathway in which specialized low-fidelity polymerases are recruited to bypass DNA lesions such as those caused by nucleoside analog incorporation, allowing DNA replication to continue [2]. BER acts by removing and replacing damaged or misincorporated nucleobases, including those introduced by nucleoside analogs [3]. BER enzymes such as DNA glycosylases recognize and excise modified bases, while endonucleases and polymerases repair the resulting abasic sites. Overexpression of both TLS and BER proteins allows cancer cells to counteract the cytotoxic effects of nucleobase and nucleoside analog-based therapies, thereby reducing treatment efficacy and promoting resistance [4,5,6]. Other pathways such as DNA mismatch repair (MMR), de novo nucleotide synthesis, and nucleobase and nucleoside analog metabolism and degradation contribute to chemoresistance [4,7,8,9]. These pathways are intricately connected, with cross-interacting mechanisms ensuring a robust and difficult-to-target development of chemoresistance [10], (Figure 1). Differential regulation of these pathways is implicated in a wide range of cancers as well as in response to many chemotherapeutic agents [11].

**Figure 2:**
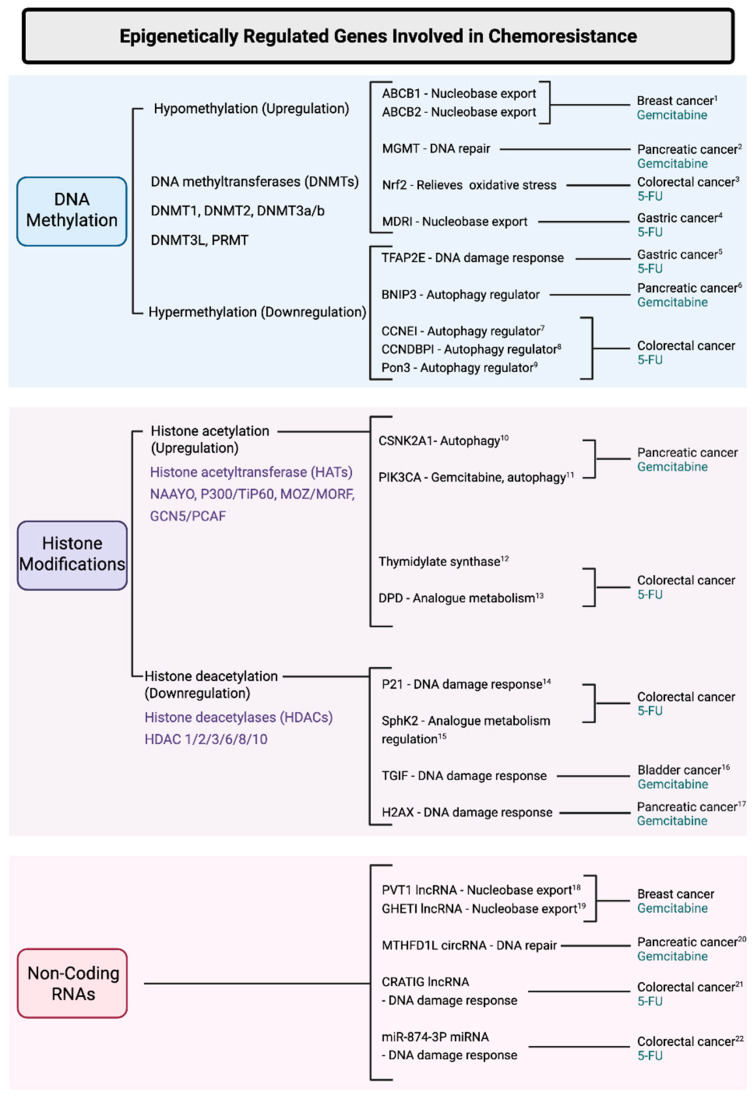
Epigenetically regulated genes contributing to nucleobase/nucleoside analog chemoresistance. Epigenetic mechanisms including DNA methylation, histone modifications, and non-coding RNA regulation play pivotal roles in modulating the expression of genes associated with chemoresistance to nucleobase and nucleoside analogs. (1) DNA methylation can lead to either hypomethylation-mediated upregulation or hypermethylation-mediated silencing of target genes. Upregulation of ABC transporters (e.g., *ABCB1*, *ABCB2*, *MDR1*) and DNA repair genes (e.g., *MGMT*, *Nrf2*, *TFAP2E*) through hypomethylation is associated with increased drug efflux and reduced genotoxic stress in various cancers, including breast, pancreatic, colorectal, and gastric cancers. In contrast, hypermethylation can suppress autophagy-related genes including *BNIP3*, *CCNE1*, *CCNDBP1*, and *Pon3*, contributing to chemoresistance by impairing cell death pathways. (2) Histone modifications also regulate chemosensitivity. Increased histone acetylation, mediated by histone acetyltransferases (HATs), promotes the expression of genes involved in autophagy (*CSNK2A1*, *CYP2C9*), analog metabolism (*DPD*, *TYMS*), and drug response (*PIK3CA*). Conversely, histone deacetylation via histone deacetylases (HDACs) can repress genes involved in DNA damage response (e.g., *CDKN1A2*, *P21*, *TGIF*, *H2AX*) and metabolism regulation (*SphK2*), further contributing to resistance in cancers treated with gemcitabine or 5-FU. (3) Non-coding RNAs, including lncRNAs (e.g., *PVT1*, *GHET1*, *CRAT16*), circRNAs (e.g., *MTHFD1L*), and miRNAs (e.g., *miR-874-3P*), influence chemoresistance by regulating nucleobase export, DNA repair, and damage response mechanisms. These epigenetically regulated ncRNAs have been implicated in resistance across multiple cancers, including breast, pancreatic, and colorectal cancers. Up-arrows indicate upregulation and down-arrows indicate downregulation. Created with BioRender.com (web application, accessed on 6 May 2025). Citations: 1 [17], 2 [18], 3 [19], 4 [20], 5 [21], 6 [22], 7 [23], 8 [24], 9 [25], 10 [13], 11 [26], 12 [27], 13 [28], 14 [29], 15 [30], 16 [31], 17 [32], 18 [33], 19 [34], 20 [35], 21 [36,37], 22 [38].

In recent years, there has been growing recognition that chemoresistance is regulated not only by genetic factors but also by epigenetic modifications—heritable changes in gene expression that do not involve alterations to the DNA sequence. Epigenetic regulation encompasses a range of mechanisms, including DNA methylation, histone modifications, and the involvement of ncRNAs, all of which contribute to the dynamic control of gene expression (Figure 2). These modifications can influence the accessibility of transcription factors to DNA, thereby modulating genes involved in chemoresistance-related DNA repair [12].

Cancer-related genes are particularly sensitive to epigenetic changes, which can either increase expression of proto-oncogenes or decrease expression of tumor suppressor genes. For example, aberrant DNA methylation patterns have been associated with decreased expression of tumor suppressor genes, while histone modifications can either promote or repress the transcription of genes conferring nucleoside analog resistance [13,14]. Furthermore, ncRNAs, including microRNAs (miRNAs), lncRNAs, and circRNAs, have been shown to play crucial roles in the post-transcriptional regulation of genes within these chemotherapeutic resistance pathways [15].

This review aims to provide a comprehensive overview of the current understanding of epigenetic contribution to nucleobase and nucleoside analog chemoresistance through the modulation of DNA repair and drug metabolism pathways. We will explore the mechanisms by which epigenetic modifications influence these pathways, focusing on the interplay between epigenetic and transcription factors. The review will also examine the roles of advanced epigenetic mechanisms, such as RNA modifications and 3D genome organization, in driving chemoresistance. Additionally, we will discuss the implications of these findings for the development of novel therapeutic strategies targeting the resensitization of resistant cancers with a focus on ductal adenocarcinoma due to its remarkable ability to quickly confer resistance to nucleobase analogs such as gemcitabine [16].

## 2. Epigenetic Mechanisms Underlying Nucleobase and Nucleoside Chemoresistance

Epigenetic mechanisms play a critical role in the development of chemoresistance by modulating the expression of genes involved in DNA repair, drug efflux pumps, drug metabolism, and apoptosis signaling. In recent years, research has uncovered the significant impact of these epigenetic processes in driving chemoresistance, revealing their essential roles in both normal cell survival and the pathogenesis of cancer and chemoresistance [39]. Epigenetic mechanisms, including DNA methylation, histone modifications, and ncRNA action, provide an additional layer of control and plasticity that allows cancer cells to respond dynamically to drugs and maintain a survival advantage under chemotherapeutic stress such as widespread DNA damage.

DNA methylation involves the action of DNMTs including DNMT1, DNMT3A, and DNMT3B to catalyze the addition of a methyl group to the 5’ position of cytosine residues within CpG dinucleotides [40]. This modification typically results in transcriptional repression, as the methylation of CpG islands inhibits the binding of transcription factors to the gene promoter regions [41]. Such modifications can also repress transcription by recruiting repressive complexes that promote chromatin condensation [41]. DNA methylation is a key mechanism in the regulation of genes involved in DNA repair and drug metabolism seen in nucleoside analog-resistant cancers, such as in colorectal and pancreatic cancer [42].

During the development of chemoresistance, multiple cellular pathways are dynamically regulated to counteract the cytotoxic effects of nucleoside analogs. These include DNA damage bypass and repair mechanisms, efflux transporters, enzymatic degradation pathways, alteration in nucleotide biosynthesis, and autophagy. For instance, hypermethylation of the promoters of human equilibrative nucleoside transporter 1 (hENT1) and deoxycytidine kinase (dCK) confers gemcitabine resistance in cervical cancer [43].

Aberrant DNA methylation is a staple of cancer tumorigenesis of resistance to several other nucleoside analogs via other pathways such as increased expression of proteins involved in DNA repair and nucleotide catabolism pathways [4]. For instance, in colorectal cancer, hypermethylation of the MLH1 gene, a key DNA mismatch repair gene, results in its silencing and contribution to chemoresistance by allowing cancer cells to accumulate mutations and evade cell death [44,45]. Similarly, in ovarian cancer, hypermethylation of BRCA1 results in impaired homologous recombination repair and diminished DNA damage response [46]. Histone modifications present another key epigenetic mechanism that influences tumorigenesis and chemoresistance. Histones, the protein components of chromatin, can undergo various post-translational modifications, including methylation, acetylation, ubiquitination, and phosphorylation. These modifications occur primarily on the N-terminal tails of histones H3 and H4 and play a crucial role in determining chromatin structure and therefore gene expression [47]. HATs acetylate histones, which typically results in transcriptional activation. Acetylation neutralizes the positive charge on histones, weakening their interaction with DNA, resulting in a more relaxed chromatin structure that allows transcription factors and transcription machinery to access DNA more easily [48]. In contrast, the removal of acetyl groups by histone deacetylases (HDACs) leads to chromatin condensation and therefore to the repression of transcription [49].

Across all types of cancer, histone modifications play a crucial role in the fast and flexible control of gene expression, which is essential for the development of resistance to nucleoside therapies. For example, upon prolonged treatment of gemcitabine in pancreatic cancer, there is a rapid increase in histone acetylation at the promoters of pro-autophagy genes such as *CSNK2A1* and analog metabolism genes such as Cytidine deaminase (*CDA*), resulting in both the degradation of gemcitabine in lysosomes as well as the conversion to inactive metabolites that are transported out of the cell [13,50]. Histone methylation, another key modification, can promote or inhibit transcription depending on the specific residues modified. For instance, the loss of trimethylation of histone H3 lysine 9 (H3K9me3), histone H3 lysine 27 (H3K27me3), and histone H4 lysine 20 (H4K20me3) is associated with the loss of transcriptional repression and is a characteristic feature of gemcitabine-resistant pancreatic cancer cell lines [51]. In contrast, methylation of histone H3 arginine 2 (H3R2me) is a marker of transcriptional activation and is often found at the promoters of genes related to chemoresistance through drug metabolism [12]. In addition, there is evidence pointing to the significant contribution of histone acetylation and methylation in the regulation of transcription factors (TFs) regulating previously stated nucleobase/nucleoside resistance pathways as well as more indirect pathways of resistance such as EMT and stemness through regulatory protein complexes [52,53,54,55].

Abnormal regulation of histone modifications has been implicated in the tumorigenesis of various cancers as well as in the emergence of chemoresistance. For instance, in gemcitabine-resistant pancreatic cancer, global hypoacetylation contributes to resistance and treatment with histone deacetylase inhibitors (HDACis) results in resensitization [51,56]. Drugs targeting histone-modifying enzymes, such as HDACis, have emerged as promising therapeutic strategies for modulating chemoresistance [57]. HDACis can restore normal histone acetylation patterns and have shown potential in preclinical models of chemotherapeutic resensitization, including gemcitabine in the treatment of pancreatic cancer [58,59].

NcRNAs have also emerged as determinants of chemoresistance to nucleoside analogs. ncRNAs include miRNAs, lncRNAs, and circRNAs, which have all been shown to be involved in regulating gene expression at the transcriptional and post-transcriptional levels. MicroRNAs (miRNAs) are small RNA molecules, typically 19–25 nucleotides long, that regulate gene expression by binding to complementary sequences in the 3′ untranslated regions (UTRs) of target mRNAs, resulting in either mRNA degradation or inhibition of translation [60]. Dysregulation of miRNAs is involved in chemoresistance through altering key pathways such as DNA repair, TLS, BER, nucleotide biosynthesis, nucleoside catabolism, and membrane transport. These processes collectively impact the effectiveness of chemotherapy agents like 5-fluorouracil (5-FU), gemcitabine, cytarabine (Ara-C), and fludarabine, which rely on proper metabolic activation and DNA incorporation to exert cytotoxic effects. For example, upregulation of miR-21 has been shown to downregulate the mismatch repair (MMR) proteins MLH1 and MSH2, resulting in 5-FU resistance in colorectal cancers [42].

LncRNAs are a varied class of ncRNAs, exceeding 200 nucleotides in length, that play key roles in gene regulation, such as chromatin remodeling, transcriptional regulation, and post-transcriptional processing [61]. Dysregulation of lncRNAs has been shown to regulate the expression of key chemoresistance genes [62,63,64,65]. For instance, the lncRNAs *PVT1* and *GHET1* have been implicated in gemcitabine resistance in breast cancer via the upregulation of cellular nucleoside exporters and autophagy modulation [66,67].

CircRNAs are ncRNAs that form covalently closed-loop structures; CircRNAs introduce an additional level of complexity to the regulation of gene expression in cancer cells. These molecules can act as miRNA sponges, binding miRNAs and preventing their interaction with target mRNAs, thus regulating gene expression [68]. For example, circ-*MTHFD1L* has been shown to sponge *miR-615-3p*, resulting in the upregulation of *RPN6*, therefore promoting DNA repair in pancreatic ductal adenocarcinoma, resulting in gemcitabine resistance [69]. The dysregulation of circRNAs has been linked to chemoresistance to nucleosides in various cancers, and their distinct structure makes them especially useful for making targeted therapeutic interventions [70].

## 3. Epigenetic Regulation of Nucleotide Biosynthesis Contributing to Chemoresistance

Cancer cell proliferation necessitates the replication and repair of DNA, and nucleotide biosynthesis is of utmost importance for this. Nucleoside analog chemotherapy resistance is often associated with the strong control of nucleotide biosynthesis regulation through epigenetics [71]. A correlation exists between the expression of some enzymes involved in nucleotide metabolism and the resistance to chemotherapy drugs like 5-fluorouracil (5-FU), gemcitabine, and cytarabine [72].

A key nucleotide biosynthesis protein, SHMT serves as a scaffold involved in the thymidylate cycle with DHFR and TS. Dynamically controlled SHMT is evidenced in cancers with 5-FU resistance, arising from dependence on thymidylate synthesis, bypassing drug activation control [73]. Histones associated with the promoter region of the SHMT gene exhibit acetylation and methylation marks. Emerging cancer therapies can induce metabolic reprogramming, such as alterations in the one-carbon pathway, resulting in decreased levels of S-adenosylmethionine (SAM), a critical methyl donor for DNA methylation. This reduction in SAM availability can result in changes to DNA and histone methylation patterns in tumor cells, influencing the efficacy of nucleoside chemotherapy treatments and resistance [42].

Moreover, deoxycytidine kinase (dCK), which is an important enzyme in the phosphorylation and subsequent activation of nucleoside analogs such as gemcitabine, is also regulated by methylation marks on DNA as well as histone changes [74]. In resistant cancer cell lines, *DCK* is downregulated via promoter hypermethylation and thus cannot phosphorylate the nucleoside, resulting in reduced cytotoxicity [64,75].

A number of transport proteins and enzymes work synergistically to control the cellular uptake, circulation, metabolism, and excretion of nucleoside analogs and their metabolites. The regulation of epigenetic pathways modulating the expression of such transporters greatly influences these processes and affects the efficacy of some chemotherapeutic agents containing nucleoside analogs such as 5-FU, gemcitabine, and cytarabine [76,77]. Moreover, the upregulation of enzymes like deoxycytidine deaminase (CDA), which deaminates nucleoside analogs such as cytarabine and gemcitabine, contributes to chemoresistance by metabolizing active nucleoside analogs to their inactive forms. These modifications that govern the expression of such enzymes allow cancer cells to bypass the regulated biogenesis of nucleotides required for DNA replication and repair. This difference creates underlying mechanisms that make the cells more resistant to chemotherapy [4,78].

### 3.1. Epigenetic Regulation of Nucleobase and Nucleoside Analog Metabolism and Transport

The entry of nucleoside drugs into cells is dependent on the presence of particular nucleoside transporters. Human equilibrative nucleoside transporter 1 (hENT1) permits passive intake, while Human Concentrative Nucleoside Transporter 1 (hCNT1) controls active transport of nucleosides into a cell. Epigenetic regulation of these nucleoside transporters impacts the therapeutic potential of nucleoside analogs [30,76]. In addition, a decreased expression of hCNT1 has also been noted in 5-FU-resistant colorectal cancer cells [79]. Moreover, efflux transporters, such as Breast Cancer Resistance Protein, BCRP, also known as ABCG2, can expel nucleoside analogs from cancer cells, which decreases their intracellular levels, thus contributing to resistance. Other efflux pumps are often controlled by histone modification and DNA methylation. For instance, P-glycoprotein (P-gp) overexpression in cancer cells is often associated with the methylation of its regulatory sites, a modification facilitated by DNMTs, resulting in enhanced susceptibility to chemotherapy [80]. Resistance mechanisms often include altered nucleoside metabolism alongside transporter regulation. DNA methylation and histone modifications can regulate the expression of dihydropyridine dehydrogenase (DPD), which is crucial for the metabolism of 5-FU. Hypermethylation of the DPYD gene promoter leads to decreased DPD and therefore reduced catabolism of 5-FU and increased cytotoxic effects, illustrating that epigenetic modifications like promoter hypermethylation may overcome chemotherapy resistance [81].

### 3.2. Epigenetic Regulation of DNA Repair Pathways by Nucleobase and Nucleoside Analogs

Nucleobase and nucleoside analogs can indirectly influence the epigenetic landscape by inducing DNA damage that triggers repair pathways that can lead to changes in chromatin structure. Certain analogs, such as azacitidine, directly affect epigenetics by inhibiting DNMTs, resulting in hypomethylation and the reactivation of silenced genes [82]. The efficient excising and bypassing of altered bases is crucial for cancer cells to withstand the treatment of nucleoside analogs. The primary pathway involved in nucleoside resistance is BER and secondly is TLS, which serves to bypass lesions that BER cannot repair [83].

BER is the repair mechanism of small DNA lesions, such as those inflicted by incorporating nucleoside analogs. Like other DNA repair mechanisms, BER is regulated by epigenetic factors such as histone modifications. Histone acetylation by Histone Acetyl Transferases (HATs) p300/CBP and GCN5 also fosters the expression of DNA polymerase β (*POLβ*), which is critical in the repair of base lesions from nucleoside analogs. In resistant cancer cells, the overexpression of *POLβ* helps to efficiently repair nucleoside analog-induced lesions on DNA, thus aiding survival and resistance [84].

The other pertinent repair pathway is TLS, where specialized low-fidelity DNA polymerases bypass replication-blocking DNA lesions [85]. Epigenetic regulation of one of the principal enzymes in TLS, DNA polymerase η (Polη), contributes to nucleoside analog resistance. Histone acetylation at the promoter region of Polη by acetyltransferase GCN5 at H3K9 may contribute to its increased expression, enabling cells to bypass analog-induced DNA lesions when they are present [86].

### 3.3. Epigenetic Regulation and Autophagy in Chemoresistance

The increase in organelle and macromolecular autophagy in cancer cells allows for the recycling of cellular structures as well as the sequestering of chemotherapeutics [87]. Moreover, preclinical studies have shown that autophagy aids in developing resistance against nucleoside analogs by increasing the degradation of these agents while permitting cell survival [88].

Various epigenetic modifications, including the addition of methyl groups to DNA and acetylation of histones, control the expression of genes associated with autophagy. The acetylation of histones serves as a hallmark of upregulation. Thus, *CSNK2A1*, a gene associated with the phosphorylation and activation of key autophagy regulators, is overexpressed in gemcitabine-resistant pancreatic cancer cells [14,30]. In addition to the above, several other pro-autophagic factors, such as *BCL2* and *BNIP3*, are proposed to have their expression elevated in cells exhibiting resistance to 5-FU and gemcitabine [89]. Through epigenetics therapies, it is possible to resensitize cancer cells by decreasing autophagy. For instance, the use of HAT p300 inhibitor C646 in combination with gemcitabine was able to decrease *CSNK2A1* expression by preventing H3K27 acetylation, resulting in decreased cell viability by over 50% compared to gemcitabine alone [1].

### 3.4. Therapeutic Strategies Targeting Epigenetic Dysregulation

**Figure 3:**
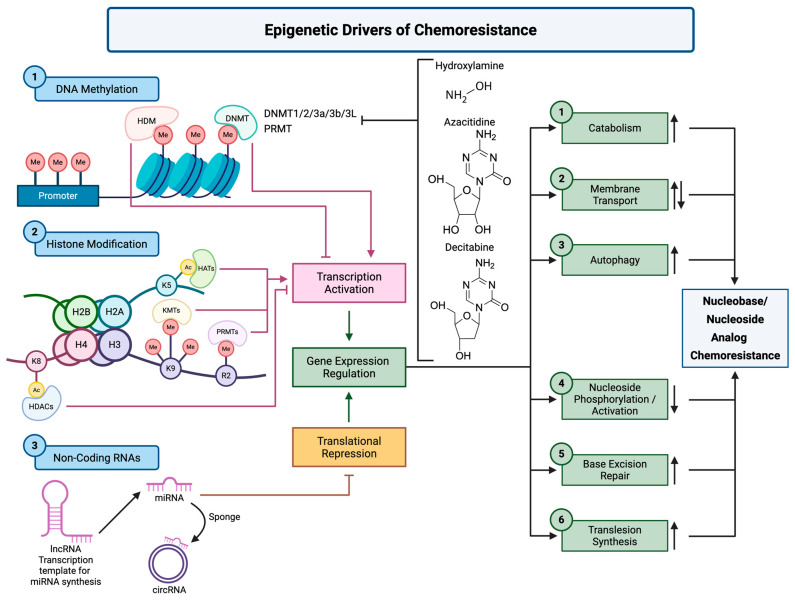
Epigenetic Drivers of Chemoresistance to Nucleobase/Nucleoside Analogs. Multiple epigenetic alterations cooperatively contribute to chemoresistance against nucleobase and nucleoside analogs by modulating the transcriptional and translational activity of genes involved in drug response. (1) DNA methylation by DNA methyltransferases (e.g., DNMT1/2/3a/3b/3L) and protein arginine methyltransferases (PRMTs) induces promoter hypermethylation and chromatin condensation, leading to transcriptional repression. Agents such as azacitidine and decitabine can inhibit DNMT activity, promoting DNA demethylation and gene reactivation. (2) Histone modification affects chromatin structure and gene expression. Acetylation via HATs and deacetylation via HDACs control accessibility, while methylation at specific lysine or arginine residues regulates transcriptional activation or repression. HDAC inhibitors like vorinostat and panobinostat can relax chromatin to restore apoptotic and DNA repair gene expression. (3) Non-coding RNAs, including lncRNAs and circRNAs, modulate translation by acting as templates or sponges for miRNAs that target mRNAs involved in drug metabolism and cell survival. These epigenetic mechanisms alter six downstream cellular processes that directly impact chemotherapeutic response. (1) Catabolism may be suppressed by restoring expression of metabolic regulators. (2) Membrane transport is impaired by epigenetic silencing of transporter genes such as *SLC29A1*. (3) Autophagy is often enhanced, promoting drug resistance through cellular adaptation. (4) Nucleoside phosphorylation/activation is suppressed due to silencing of kinases such as dCK. (5) Base excision repair is inhibited, limiting the removal of damaged bases. (6) Translesion synthesis (TLS) is stimulated, allowing replication despite DNA lesions. Collectively, these alterations compromise intracellular drug activation and reduce the therapeutic efficacy of nucleobase and nucleoside analogs. Up-arrows indicate upregulation and down-arrows indicate downregulation. Created with BioRender.com (web application, accessed on 6 May 2025).

The emerging recognition of the possibility of treating chemoresistance through therapies targeted at resensitizing cancer cells has led to therapies trying to undo biological changes and make them responsive to nucleoside analogs (Figure 3). Such therapies that target the epigenetic mechanisms, including but not limited to methylation of DNA, modifications of histones, and ncRNA, show significant potential [90].

### 3.5. DNA Methyltransferase Inhibitors

One of the functions of DNA methylation is the modification of gene expression by altering transcription. Aberrant DNA methylation of genes involved in drug metabolism and apoptosis suppresses their expression, contributing to chemoresistance. DNA methyltransferase inhibitors (DNMTis) such as decitabine have been shown to reverse these epigenetic silencing events and restore tumor sensitivity to agents in resistant cancer models [91,92]. DNA methyltransferase inhibitors function as nucleoside analogs, azacitidine, guadecitabine (SGI-110), RX-3117, 5-fluoro-2′-deoxycitidine (FdCyd), 5,6-dihydro-5-azacytidine (DHAC), and cladribine. Nucleoside DNMTis are incorporated into DNA during replication, where they form covalent bonds primarily with DNA methyltransferase 1 (DMNT1) and lead to enzyme degradation and passive DNA methylation. Other DNMTis, referred to as non-nucleoside DNMTis, act without DNA incorporation, as observed with compounds that inhibit activity by directly binding to DNMTs, suppressing DNMT expression, or interfering with methyl donor availability. DNMTis can reactivate repressed tumor suppressor genes and resensitize cancer cells to conventional chemotherapy. For instance, azacitidine has been tried clinically with other nucleoside analogs, namely 5-FU, for colorectal cancer patients and has shown an ability to resensitize 5-FU-resistant tumors [93]. Research indicates that azacitidine can potentially enhance the efficacy of 5-FU on cancer cells by counteracting the methylation-induced silencing of the DNA repair pathways crucial for the development of resistance to nucleoside analog treatment [94]. This approach may also benefit other types of cancer where the methylation of genes such as *SLC29A1* and *DCK* is pivotal for the uptake and activation of nucleoside analogs [95,96].

Even so, using DNMTis in preclinical models and early-phase clinical trials has raised concerns about the potential of off-target impacts and the intricate nature of tumor heterogeneity when using them in combination strategies due to their chemical and metabolic instability [97,98]. Azacitidine derivatives including CP-4200 and zebularine, decitabine derivatives such as NPEOC-DAC, and thio-cytidine derivatives T-dCyd and 5-aza-5-dCyd were developed to address this challenge [77,98]. For example, zebularine induces promoter hypomethylation of tumor suppressor genes such as *p15* and *p57* by stabilizing DNMT-DNA binding and shows highly selective DNMT1 inhibition with low toxicity [77,98].

### 3.6. Active DNA Demethylation

Active DNA demethylation refers to a replication-independent enzymatic process primarily mediated by the ten-eleven translocation dioxygenase (TET) family of dioxygenases, which catalyzes the oxidative conversion of 5-methylcytosome (5mC) to essential intermediates such as 5-hydroxymethylcytosine (5hmC), 5-formylcytosine (5fC), and 5-carboxylcytosine (5caC). These intermediates are then recognized and excised by DNA glycosylase (TDG), which initiates the BER pathway, replacing modified cytosine with unmodified cytosines. Additional regulatory proteins such as GADD45A and GADD45B further facilitate the demethylation process by recruiting BER machinery and coordinating chromatin remodeling. As active DNA demethylation is crucial for tumor suppression, dysregulation of this process contributes to carcinogenesis and chemoresistance by promoting abnormal gene expression or silencing [99].

Impaired DNA demethylation contributes to chemoresistance by maintaining abnormal methylation of critical genes involved in drug uptake, activation, and apoptotic response. Loss of function mutation in genes coding for TET and TDG leads to persistent epigenetic silencing, thereby reducing the efficacy of nucleoside and nucleobase analog-based therapies [100,101].

Among the genes coding for TET family enzymes, reduced expression of *TET1* and subsequent loss of 5hmC contribute to stable silencing of tumor suppressor genes such as *BRCA1* and *PTEN* in breast cancer. This silencing impairs apoptotic and DNA damage response pathways and facilitates the development of resistance to chemotherapeutic agents including tamoxifen and platinum compounds. At the molecular level, the loss of *TET1* promotes the recruitment of HDAC repressor complexes, reinforcing transcriptional silencing and epigenetic rigidity in resistant cancers [101]. TET2 is frequently mutated or functionally suppressed in both lymphoid and myeloid malignancies. In myeloid cancers such as AML and MDS, loss of TET promotes clonal expansion and blocks differentiation, while in lymphoid malignancies like AITL and DLBCL, TET activity is often impaired even without mutation, due to metabolic factors such as 2-hydroxyglytarate accumulation. 

### 3.7. Histone Deacetylase (HDAC) Inhibitors

Transcriptional repression and chromatin condensation are the result of the removal of acetyl groups from histones through the actions of HDACs. The action of HDAC inhibitors promotes increased gene transcription by relaxing the tightly coiled helical structure of DNA. Euchromatin has also been recognized as a marker for apoptosis and DNA repair. HDAC inhibitors have also shown the ability to modulate the expression of chemoresistance genes associated with nucleoside analogs. In preclinical studies, the use of HDAC inhibitors has shown the ability to resensitize some cancer cells particularly to nucleoside analogs by disengaging the silencing of apoptotic genes and modulating the expression of DNA repair proteins [102,103]. HDAC inhibitors are categorized into several structural classes, each with distinct molecular targets and clinical advantages. Hydroxamic acids, such as vorinostat and panobinostat, are potent pan-HDAC inhibitors widely studied in hematologic cancers. Short-chain fatty acids like valproic acid act more broadly but with lower potency. Benzamides such as entinostat selectively inhibit class I HDACs, while cyclic tetrapeptides like romidepsin target class I HDACs with high specificity. Sirtuin inhibitors such as nicotinamide and EX-527 modulate NAD+-dependent HDACs and are under investigation for cancer [102]. For example, combining vorinostat with gemcitabine has shown considerable clinical success in overcoming the epigenetic repression of key drug transporters such as hENT1 in gemcitabine-resistant pancreatic cancer [101]. While preclinical and early-phase clinical investigations have yielded optimistic results, the clinical application of HDAC inhibitors in oncology is limited by adverse effects, such as gastrointestinal toxicity and myelosuppression [51]. Furthermore, their lack of selectivity and transient pharmacological effect in vivo requires the development of more particular and tolerable formulations. As a result, the combination of HDAC inhibitors with nucleoside analogs or other epigenetic drug is an active area of study [102].

### 3.8. Non-Coding RNA Modulators

Another relevant aspect in the development of chemoresistance is the activity of ncRNAs. MiRNAs, lncRNAs, and circRNAs modulate the expression of the genes responsible for DNA damage response, cellular transport, and cell survival [104,105,106].

Targeted therapies aimed at miRNAs include restoring the expression of downregulated suppressor miRNAs or genomic alterations involving CRISPR [107,108]. Thus, ncRNAs serve as another potential avenue available to be targeted in order to overcome or combat chemoresistance.

miRNA mimics can also be used in conjunction with nucleoside analogs to increase expression of regulating genes such as *miR-21*, which leads to 5-FU resistance [109,110]. Regarding the research conducted by Li et al., in 2020, it was highlighted that miRNA-34a coadministrated with gemcitabine in pancreatic cancer cells led to enhanced apoptosis alongside reduced tumor size by downregulating key targets such as *BLCL2*, an anti-apoptotic gene, and *NOTCH1*, a regulator of cancer stemness and survival [111]. Furthermore, lncRNA-based therapies have also shown promise for the treatment of resistance to nucleoside analogs. For instance, lncRNA *PVT1* has been implicated in gemcitabine resistance in breast cancer by upregulating drug efflux transporters and autophagy-related genes, thereby decreasing intracellular gemcitabine accumulation and enabling cancer cells to evade gemcitabine-induced apoptosis [66,112]. *PVT1* can induce gene silencing with small interfering RNA (siRNA) containing antisense oligonucleotides, enhancing clinical outcomes through autophagy-mediated resistance reversal to nucleoside analogs [113,114,115].

Moreover, circRNAs like circ-*MTHFD1L* have been shown to modulate the process of nuclear DNA repair and mediate resistance to nucleoside analogs. The modulation of synthesis pathways that form circRNA by RNA-based therapeutics may provide new alternatives for overcoming resistance by altering DNA repair in cancer cells [116].

### 3.9. Combination Therapies

The use of epigenetic drugs, in addition to nucleoside analogs, presents an innovative strategy for improving cancer outcomes and preventing relapses. Implementing DNMT and HDAC inhibitors alongside nucleoside analogs has yielded promising results in preclinical models [117]. These studies suggest that the proposed combinations could help reawaken sensitivity to chemotherapy by reversing epigenetic tumor suppressor silencing and modulating expression of relevant drug metabolism, repair, and apoptotic pathways. For instance, research in pancreatic cancer demonstrates that the coadministration of HDAC inhibitors and gemcitabine enhances *SLC29A1* and *DCK* gene expression, which are integral to gemcitabine transport and activation [30].

These combination treatments are particularly effective for increasing the clinical benefits of therapies relying on nucleoside analogs in cancers that have developed acquired resistance. However, as discussed, an aggressive approach to optimizing dose and scheduling will be critical for striking a balance between maximizing therapeutic efficacy and minimizing adverse effects.

## 4. Disease Focus: Overcoming Tumor-Specific Resistance to Nucleobase/Nucleoside Analogs

### 4.1. Pancreatic Cancer

Considered one of the most aggressive forms of cancer, resistant pancreatic cancer, particularly to nucleoside analogs such as gemcitabine, poses a relentless clinical challenge [118]. Some of the markers of gemcitabine-resistant pancreatic cancer include decreased nucleoside cellular transport, modification of the catabolism pathways, and heightened ability of DNA repair with a decreased response to damage.

It is customarily outlined that the lack of expression of human equilibrative nucleoside transporter 1 (hENT1) leads to resistance to gemcitabine. As emphasized before, hENT1 is a vital component in the transport of gemcitabine into the cell, and thus aids in the development of chemoresistance. Patients afflicted with pancreatic cancer are known to become resistant due to a very penetration of gemcitabine into the cell [51]. Gemcitabine deactivation can be attributed to the altered expression of HDACs, which has been shown to upregulate autophagy-related genes responsible for the destruction of gemcitabine [30,119]. Furthermore, the ability for gemcitabine-resistant pancreatic cancer cells to survive gemcitabine-inflicted DNA damage is polymerase η. This specialized polymerase, alongside several others in the y-family of polymerases, can bypass lesions in DNA by inserting nucleotides across from gemcitabine or other DNA damage prevention in a process called TLS, allowing for continued DNA replication and therefore pancreatic cancer cell proliferation [120,121,122]. A combination of avalanche inhibitors and inhibition of TLS polymerases was proven to deliver dependable results in preclinical pancreatic cancer trials [30].

Recent research has also uncovered epigenetic mechanisms such as DNA methylation that silence DNA damage repair genes like BRCA1 and BRCA2. This loss of repair capacity impairs homologous recombination, ultimately contributing to resistance against nucleoside analogs like gemcitabine [123].

### 4.2. Colorectal Cancer

Another type of cancer that is commonly treated with nucleoside analog therapy, particularly 5-FU, is colorectal cancer (CRC). The use of 5-FU is frequently met with resistance, which greatly limits the value of the drug’s treatment potential. One of the primary factors contributing to resisting the drug is alteration in the DNA repair pathways, especially mismatch repair (MMR). In CRC, hypermethylation-induced silencing of the MLH1 gene, which is critical for mismatch repair, is frequently observed in chemoresistant tumors, contributing to genomic instability and reduced sensitivity to chemotherapy [107,124].

Along with MMR deficiencies, the role of autophagy in resistance to 5-FU in CRC has emerged as an area of interest. Research on the epigenetic modulation of autophagy genes such as *BNIP3* and *BCL2* has demonstrated that these genes enable the survival of CRC cells treated with 5-FU [125]. A lot of effort has been devoted towards 5-FU and HDAC inhibitor combinations aimed at overcoming the autophagy-based resistance.

In preclinical studies, the HDAC inhibitor vorinostat in combination with 5-FU led to enhanced apoptosis, in tandem with diminished levels of autophagy in 5-FU-resistant CRC cells [56]. The overall decrease in expression of the hENT1 and dCK drug transporters vital to 5-FU’s metabolism enhances resistance in CRC. Additionally, it has been observed that the effectiveness of the drug is reduced by epigenetic silencing via the methylation and histone modification of these genes [79,126,127].

### 4.3. Breast Cancer

The overwhelming majority of breast cancers tend to quickly become unresponsive to nucleoside/nucleobase analog drugs such as 5-FU and gemcitabine [128]. As with other treatments, chemoresistance to nucleoside analogs is commonly marked by Triple-Negative Breast Cancer (TNBC) heterogeneity with distinct and increasingly treatment-resistant tumors. Some of the treatment resistance mechanisms in TNBC include alteration in DNA repair pathways, altered cellular transport, and modulation of apoptosis and autophagy pathways. TNBC also undergoes critical epigenetic modifications, including the alteration in several genes that participate in DNA repair such as the BER and TLS pathways. In resistant breast cancer cells, polymerase η becomes upregulated, allowing these cells to survive by bypassing DNA lesions caused by nucleoside analogs [129]. Suppression of TLS polymerases using small molecules has been shown to restore sensitivity in preclinical models [130]. Apart from DNA repair, ncRNAs contribute to nucleoside analog resistance in breast cancer. The lncRNA gene *PVT1* has also been shown to actively regulate drug export and modulate autophagy, accounting for its upregulation in gemcitabine-resistant breast cancer cells [66]. Targeting *PVT1* with RNA-based therapeutics may reverse resistance by inhibiting drug efflux and modulating autophagy. Histone modifications are also a contributing factor to the development of resistance in breast cancer [110,111,131]. In the context of breast cancer, the use of HDAC inhibitors has shown potential for increasing the susceptibility of cell lines to nucleoside analog therapy by reversing the epigenetic suppression of important drug efflux pumps such as hENT1 and dCK [56]. There are clinical trials with the use of HDAC inhibitors combined with nucleoside analogs, and preliminary studies indicate that the combination of these two therapies may enhance treatment outcomes in advanced stages of drug-resistant breast cancer.

## 5. Therapeutic Strategies, Challenges, and Future Directions in Epigenetic Research

### 5.1. Innovative Epigenetic Drugs

Some of the more recent therapies aim to circumvent the drug resistance posed by nucleoside analog therapies through epigenetics. Controlled research into the role of epigenetics in cancer along with better understanding of how nucleoside analogs enable the development of new strategies designed to overcome the resistance mechanisms posed by these drugs. Focusing on epigenetic modifiers such as DNMT and HDACis alterations to the epigenetic landscape can be studied [132,133,134].

Invasive myelodysplastic syndromes along with AML are now being treated in the clinic with the DNMTi drugs azacitidine and decitabine [135,136]. Other emerging treatments target the enzymes that lead to the modification of histone and, thus, the chromatin structure like HDACs and other chromatin-interacting proteins.

Vorinostat and romidepsin, like other HDAC inhibitors, are also being investigated for their ability to alter histone acetylation and resensitize tumors to nucleoside analogs. These inhibitors may activate pro-apoptotic gene expression while returning drug efflux transporter expression to regular levels, which makes cancer cells more vulnerable to nucleoside analogs [137,138]. For example, in one study with preclinical models of gemcitabine-resistant pancreatic cancer, HDAC inhibitors showed the ability to significantly restore sensitivity to gemcitabine by unsilencing crucial drug influx transporters [115]. Although these epigenetic drugs hold great potential, there is still limited clinical application due to off-target effects, general toxicity, and the complexity of tumor-specific changes in epigenetics. Greater efficacy in the clinic will require improvement in drug specificity and delivery.

### 5.2. CRISPR-Based Epigenome Editing

The advent of CRISPR technology has transformed genetics and is now being extended to epigenetics for direct alteration in the epigenome. CRISPR/Cas9-based epigenome editing systems enable the simulation of a wide range of epitomic features, such as the following: methylation of DNA, modification of histones, remodeling of chromatin, and more. The ability to add or remove epigenetic marks from defined areas of the genome can be performed by utilizing a dead Dcas9 (dCas9) with epigenetic markers such as DNMTs or HATS. An example of this is the use of CRISPR to demethylate *SLC29A1* and *DCK* promoter regions, increasing their expression, resulting in resistant cancer cells becoming resensitized to nucleoside analogs [139]. In other studies, CRISPR systems have also been used to histone acetylate the promoters of some DNA repair genes, which resensitizes cancer cells to chemotherapy [140].

The above studies represent the cutting edge in researching CRISPR-based epigenome editing technology. However, some barriers remain in using this technology in clinical practice effectively. These barriers include the potential collateral damage epi-editing could inflict, targeting the specificity of the delivery system for the CRISPR. Regardless of these challenges, rescripting epigenetic modifications to combat the resistance to chemotherapy may become of the prime capabilities of CRISPR technology.

### 5.3. Personalized Medicine Approaches

With the continuous improvement in understanding the molecular and epigenetic world of cancer, the personalized medicine approach is advancing when it comes to managing chemoresistant cancers. Tailor-made epigenetic therapy focuses on individual tumors with specific epigenetic markers, allowing for exact targeting of chemoresistance mechanisms.

The combination of epigenetic profiling with next-generation sequencing (NGS) technologies has allowed patients’ specific epigenetic modifications associated with chemoresistance to be defined. A tumor’s DNA methylation, histone modification patterns, and ncRNA expression profile can provide insight into the epigenetic mechanisms driving resistance, enabling physicians to tailor suitable epigenetic treatments [66,133]. As an example, *SLC29A1* and *DCK* silencing via DNA methylation in some gemcitabine-resistant pancreatic cancer patients could be targeted with a pharmaco-epigenetic approach using DNMT inhibitors alongside gemcitabine [30]. Similarly, nucleoside analog-resistant tumors exhibiting BRCA1/2 methylation might respond well to those analogs when used with DNA damage response inhibitors [141].

Personalized therapies based on epigenetics can also include ncRNAs. By examining the expression profiles of miRNAs and lncRNAs in the tumors, particular resistance-associated ncRNAs can be discovered. For instance, in colorectal cancer, miR-21 overexpression upregulates several DNA repair proteins, contributing to resistance to 5-FU [142]. A personalized regimen for overcoming this resistance could be developed through miRNA mimics or inhibitors of miRNAs.

### 5.4. Challenges in Epigenetic-Based Therapies

The use of epigenetic mechanisms to address the problem of chemotherapy resistance has some potential, but achieving successful clinical application will need to overcome the following challenges:

Tumor Heterogeneity. Different tumors show great scope of epigenetic diversity and changeable marks within a tumor. This diversity is a haphazard factor in the considerably more difficult systematic approach of integrating the vision of precision medicine.

Off-Target Effects. The class of epigenetic drugs, including DNMT and HDAC inhibitors, has off-target impacts that modify the expression of undesired genes [137]. Such processes are detrimental and produce non-selective changes in normal cells, reducing the treatment gap for these agents.

Drug Resistance. Similar to conventional chemotherapy, epigenetic therapy drug resistance in cancer cells is possible. For instance, cancer cells could initiate other alternative pathways for repairing the DNA and change their epigenetic modifications, causing a resurgence in resistance [86].

Delivery and Bioavailability. The effective delivery of epigenetic drugs and CRISPR components to the tumor site is still a primary challenge [143]. More sophisticated drug delivery systems like nanoparticles or viral vectors are being investigated for better targeting and delivery of these therapies.

The development of therapies based on epigenetics has the potential to circumvent the challenges posed by chemotherapy and enhance the effectiveness of treatments using nucleoside analogs. With further research and clinical trials, these methodologies will be optimized to discover the best methods for integrating epigenetic changes into cancer treatment plans.

## 6. Conclusions: Summary of Key Findings, Clinical and Research Implications, and Future Directions

### 6.1. Summary of Key Findings

Elucidating the underlying epigenetic features that drive chemoresistance in the context of nucleoside and nucleobase analog therapies provides a new paradigm indicating how cancer cells circumvent chemotherapy. Among the major modulatory epigenetic mechanisms in cancer, DNA methylation, histones modifications, and ncRNAs play fundamental roles in controlling the expression of proteins [66,144], responsible for numerous pathways such as drug metabolism, autophagy, cellular transport, and DNA repair, which all have a direct impact on resistance development.

### 6.2. This Review Serves to Highlight the Following Key Points

DNA Methylation. The resistance to nucleoside analogs is significantly linked to the hypermethylation of *SLC29A1*, *DCK*, and *MLH1*. Decreased expression due to the hypermethylation of the promoter of these genes renders chemotherapy inefficient through lowering drug influx, drug activation, and diminishing DNA repair pathways [51,145].

Histone Modifications. The expression of genes coding for proteins involved in DNA repair and autophagy are controlled by histone acetylation and methylation. In the case of gemcitabine-resistant pancreatic cancer, the resulting increased autophagy and reduced catabolism and nucleoside analog activation pathways fuel resistance to the drugs [88].

Non-Coding RNAs. The change in expression of miRNAs, lncRNAs, and circRNAs is associated with modulating resistance pathways. *MiR-21*, *PVT1*, and circ-*MTHFD1L* have been reported to alter DNA repair, autophagy, and drug metabolism, all of which are critical in the development of chemoresistance [66].

Targeting Epigenetic Mechanisms. Therapies that utilize DNMT or HDAC inhibitors that are based on epigenetic markers are beginning to emerge as strategies to overcome chemoresistance. Combination treatment with epigenetic drugs and nucleoside analogs shows the ability to effectively resensitize cancer cells to chemotherapy, particularly in pancreatic and colorectal cancers [56,146].

Personalized Medicine. Developing next-generation sequencing (NGS) and epigenetic profiling technologies has been a step towards developing personalized epigenetic therapy. Detection of specific changes within a patient’s epigenome that lead to chemoresistance enables the design of targeted therapies to treat such resistance and improve treatment outcomes [65].

### 6.3. Clinical and Research Implications

The increasing scrutiny of the role of epigenetics in chemoresistance highlights the need to find new methods for overcoming the resistance posed by nucleoside analogs. Epigenetic modifications performed by DNMT and HDAC inhibitors and CRISPR editing technologies can alter the epigenome, making them clinically viable and could improve the efficacy of current chemotherapy regimens.

The aforementioned approach has implications for direct medical service delivery and clinical practices. This was particularly sought in the case of patients suffering from advanced pancreatic, colorectal, and breast cancers, as it was sought for curing the remaining metastases that were unresponsive to the traditional chemotherapy and radiotherapy. There is ongoing research involving the use of DNMT and HDAC inhibitors, and some of the preliminary results suggest that with such treatment, there might be an increased response to chemotherapy, most particularly when utilized together with conventional cytotoxic drugs.

Nonetheless, there are issues that need to be addressed before these conclusions are brought into routine clinical practice. As the unidimensional nature greatly lacks in the fundamental theory supporting the various classes of tumors, much more divergent approaches have to be formulated for each case with specific guidelines for targeting specific changes associated with epigenetic alterations [147]. In addition, the unexplained destructive aspects and pseudo stratification epigenetic therapies are significant and require additional investigation regarding the treatments employed, the treatment’s timeline, and concentration of dosage.

What remains a primary concern is the development of resistance to epigenetic therapies. As with classic chemotherapy, cancer cells are likely to circumvent epigenetic therapies through some form of pathway bypass, which strongly suggests the need for further work on combinational therapy approaches or new epigenetic modulators.

### 6.4. Future Directions

Research on treating cancer chemoresistance through the use of epigenetic therapies should focus on the following directions:

Combination Therapies. Adding epigenetic drugs to classical chemotherapy or targeted immune checkpoint inhibitor therapies may help overcome chemoresistance. In treating resistant pancreatic cancer models, adding HDAC inhibitors with gemcitabine has been shown to reverse resistance and other cancers may benefit from such combinations [59].

CRISPR-Based Epigenome Editing. The invention of CRISPR/Cas9 epigenome editing tools allows researchers to change the epigenome with greater specificity. This kind of technology could directly reverse the silencing of drug transporters, DNA repair genes, as well as apoptosis-promoting genes, which would render these tumor cells more susceptible to nucleoside analogs [4,148]. The major obstacle to delivering CRISPR-based therapies efficiently may be resolved in the future, especially with the rapid progress of gene editing technologies intended for clinical use. 

Non-Coding RNAs. The alteration in expression of miRNAs, lncRNAs, and circRNAs is linked with fine-tuning resistance pathways. As delineated by Zhou et al. (2020), MiR-21, PVT1, and circ-MTHFD1L have been shown to modify the intricacies of chemoresistance mechanisms concerning the changes in DNA repair, autophagy, and drug metabolism [67].

Predictive Biomarkers of Epigenetic Modification. It will be critical for personalizing treatment plans to identify precise biomarkers related to epigenetic alterations associated with chemoresistance. These biomarkers may also aid in response prediction, enabling clinicians to make optimal therapeutic decisions.

## 7. Final Reflection

This review examined epigenetic factors’ role in developing resistance to therapies based on nucleobase and nucleoside analogs. Certain forms of epigenetic modification like DNA methylation, histone modification, and ncRNA expression regulation enable cancer cells to withstand the treatment of nucleoside analogs, making treatment more difficult. The use of epigenetic therapies such as DNMT and HDAC inhibitors or even CRISPR-based editing of the epigenome has the potential to improve the effectiveness of nucleoside analogs in cancer treatments. Focus on the personalization of epigenetic therapies, design of new ncRNAs, and combination treatments should be prioritized to enhance the development of these therapies for clinical use. There is hope for the effect and precision of cancer treatment as the advancements in epigenetic profiling and CRISPR technologies continue the ever-evolving fight against chemoresistance of nucleobases and nucleosides therapies.

## Data Availability

No new data were created or analyzed in this study. Data sharing is not applicable to this article.
